# Verification of Xiphoid Point as a New Measuring Point for Chest Shape Measurement

**DOI:** 10.1155/2022/1240514

**Published:** 2022-08-25

**Authors:** Yulin Zhao, Dingbang Luh, Yue Sun, Fei Sun

**Affiliations:** School of Art and Design, Guangdong University of Technology, Guangzhou, China

## Abstract

The measurement point of chest shape based on non-rigorous ergonomics is the key problem affecting the accuracy of measurement results. Xiphoid point, as a representative human skeleton point, can provide a theoretical basis for the development of new chest shape measurement methods and technology. In order to verify the stability of xiphoid point, 30 subjects were selected to measure the chest shape based on upper limb movement in the transverse plane of human body. The displacement of mastoid and xiphoid points in the sagittal plane, coronal plane, and axial plane was compared, and the factors influencing the displacement of mastoid and xiphoid points were analyzed. The results showed that the displacement of the mammary point was the largest in the coronal plane during the upper limb movement. The displacement of xiphoid point is far less than that of mastoid point. The displacement of the breast point is closely related to the chest circumference when the upper limb is in the horizontal adduction. There was a positive correlation between the displacement of xiphoid point and the amplitude of upper limb movement. Compared with mammary point, xiphoid point is a fixed and stable measurement point of chest shape.

## 1. Introduction

Adolescence is a critical period of female breast development. Appropriate bra products play a key role in the physical health of their cardiopulmonary activities and the mental health of peer communication [1]. However, relevant literature points out that 80%–85% of girls choose inappropriate underwear [2]. Through actual interviews and analysis of the status quo of girls' underwear patents and commodities, it is found that between the unprotected waistcoat circumference and the adult bra, there is not only a lack of supplies needed to protect and accompany the growth of girls but also a lack of measurement technologies and methods and tools for the appropriate selection of products. The key lies in the most fundamental problem of the measurement point of breast shape, that is, unstable breast spot and approximate upper and lower chest circumference [3].

In ergonomics, in order to obtain the correct human body size, the necessary measurement base points are marked on the human body before measurement to indicate the correct measurement position [4]. The measurement base points usually take the protrusions of bones, joint ends, cutting marks, and sutures that can be obtained from the touch of the skin surface as the measurement basis [5–7]. The position of the measurement points is wrong, and the data of the test samples are suspicious or even wrong.

To sum up, the reasonable, accurate, and ergonomic measurement base point should be the human skeleton point, which has the characteristics of representativeness and stability. However, according to the description of human anatomy, the breast is the only female organ without bone support, and the interior is mainly gland, breast lobule, and adipose tissue [8–12]. Due to the particularity of breast tissue, breast points or human surface feature points are often extracted as the measurement points of breast shape measurement rather than the bone base points [9]. Breast point and feature point are not fixed bone points, so they are not suitable human factor measurement base points and do not conform to the basic principles of measurement [13]. In addition, it is easy to displace the breast point due to its own action or the compression of the measuring tool makes the breast deform or change the curvature, resulting in measurement errors and affecting the accuracy of chest measurement [14–17].

The breast needs to be protected and supported during the development of girls. The stable bone can be used as a fulcrum to exert force, so that the bra can stabilize the vibration of the chest [18]. The xiphoid point is located at the sternoclavicular joint, in the center of the human body, and easy to touch, so it is representative. In order to verify whether the xiphoid point conforms to the theoretical characteristics of the stability of the measuring point in ergonomics, this paper aims to compare and analyze the displacement of the xiphoid point and the breast point during the upper limb movement, so as to prove the progress of the xiphoid point as a new chest shape measuring point. It lays the foundation for the following new chest measurement methods and measurement technologies and makes the bra industry have innovative development.

## 2. Experimental Design

### 2.1. Participant

Thirty women volunteered to participate in the experiment, and none of them were pregnant or breast-feeding. As shown in Tables 1 and 2, the age, breast size, and physical characteristics of participants vary. The breast size of each participant is classified according to the existing underwear model classification method, which is based on the difference between the upper chest circumference and the lower chest circumference [19]. The purpose of the experiment is to test the stability of the new measuring point, that is, to compare the displacement of the breast point and the xiphoid point during self-measurement with the displacement of the three upper limb states of the human body and their influencing factors.

### 2.2. Participant

The breast is a three-dimensional organ. One camera cannot capture the displacement of the human body in three axes. Therefore, three Canon EOS 800D digital cameras (24.2 million effective pixels, 128G SD card) are used to record the activities of the breast in three angles of the upper limb. The three digital cameras were fixed at 0.9m from the front, left, and top of the subject. All three cameras were fixed with T-170 SLR tripods, and the focal length of the camera was adjusted with the main target in mind, so the lens needed to be adjusted at the same height as the nipple to capture the displacement of the nipple point and the new measurement point in three directions.

The experiment was completed in the laboratory where the room temperature was (25 ± 2)°C, the relative humidity was (65 ± 5)%, and the wind speed was less than 0.1 m/s, which ensured the stability of the experimental results. During the experiment, the electric lights were fully turned on to obtain sufficient lighting.

### 2.3. Test Displacement Action

**Figure 1:**
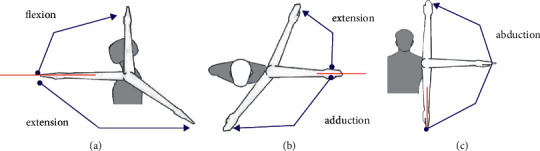
Test action of displacement experiment.

As for the selection of test movements, the upper limb activity is mainly the movement of shoulder joint, which is a complex structure composed of sternoclavicular joint, clavicle, acromioclavicular joint, scapula, glenohumeral joint, proximal humerus, and scapular chest wall joint. The movement of the acromioclavicular joint may include the movement of the clavicle relative to the scapula in three directions, namely, the abduction of the coronal axis, the anterior and posterior lifting of the sagittal axis, and the adduction and extension of the transverse axis. Among them, the range of motion of the sagittal axis is about 120°–180°, and the range of motion of the sagittal axis is about 40°–60°. The range of abduction motion of the coronal axis is 0°–180°. The range of adduction motion of the transverse axis is about 0°–135°, and the range of extension motion is about 0°–30°. As shown in Figure 1, in the milk point displacement test, the maximum range of motion of the coronal axis, the sagittal axis, and the transverse axis is selected, respectively, that is, the abduction of the coronal axis is 90° (recorded as CP1) and 180° (recorded as CP2), the elevation of the sagittal axis is 90° (recorded as SP1) and 60° (recorded as SP2), and the adduction of the transverse axis is 135° (recorded as TP1) and the extension of the transverse axis is 30° (recorded as TP2). According to the classification of joint anatomy, joints are divided into immobile joints, inching joints, and moving joints. The xiphoid point on the sternum belongs to immobile joints and is stable, but the skin covering the xiphoid point will also shift due to the influence of upper limb movement, thus affecting the accuracy of chest shape measurement. Therefore, in the selection of xiphoid point displacement measurement action, the 90° abduction (CP3) and 180° abduction (CP4) of the coronal axis with the greatest displacement effect were selected.

## 3. Experimental Process

### 3.1. Experimental Procedure

Before the test, the subjects need to bare their upper body, expose the umbilicus, and cover their hair with a mesh headgear after the messenger's hair is tied up as required. The test position is upright sitting position, which can reduce the involuntary shaking of the human body. The subjects' eyes look straight ahead, the left and right thighs are roughly parallel, the knees are bent roughly at a right angle, and the feet are flat on the ground. Before measurement, use a marker to mark the position of the breast point and xiphoid point, adjust the measurement posture of the subject, and do the specified test action. The experimenter takes pictures and stores images.

### 3.2. Displacement Measurement

**Figure 2:**
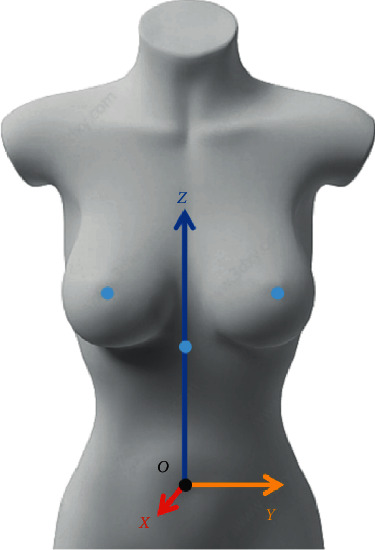
Establishing coordinate axis.

As shown in Figure 2, the geodetic coordinate system is established before the displacement results are measured. The umbilicus point is less affected by the activities of the upper limbs, so it is selected as the origin O. The coronal section of the human body is taken as the *X* axis, the direction of the x-axis is positive forward, the sagittal section is taken as the *Y* axis, the direction of the y-axis is positive leftward, the transverse section is the *Z*axis, and the direction of the z-axis is positive upward.

The displacement is measured by CorelDRAW 2019 computer software. The purpose of using this software is to have the function of measuring tool, which can mark the size of objects and the distance between objects. When measuring, turn on the snap function in the function list, select the snap object and dynamic auxiliary line, and assist in the alignment and selection of measurement in this study. In this study, the measurement style is decimal, the measurement accuracy is two decimal places, and the measurement unit is mm.

### 3.3. Statistical Analysis

The displacement of the breast point in the sagittal, coronal, and transverse planes of the human body and their respective *x*-axis, *y*-axis, and *z*-axis when performing the six actions of 90° forward lift and 60° backward lift, 135° inward retraction and 30° outward extension, and 90° outward abduction and 180° is compared with the displacement of the new measuring point in the coronal *z*-axis when performing the two actions of 90° outward abduction and 180° through descriptive statistics such as minimum value, maximum value, mean value, and standard deviation, so as to know the direction and axial direction of the maximum displacement.

In order to analyze the factors affecting the breast point and the new measuring point except for the upper limb movements, the Pearson correlation between the breast point and the new measuring point and the test value sig with significant difference were tested. The statistical significance was compared with 0.05 and 0.01. If it was greater than 0.05 or 0.01, the difference was significant. All analyses were performed using SPSS 25.

## 4. Experimental Analysis

### 4.1. Nipple Displacement Analysis

**Figure 3:**
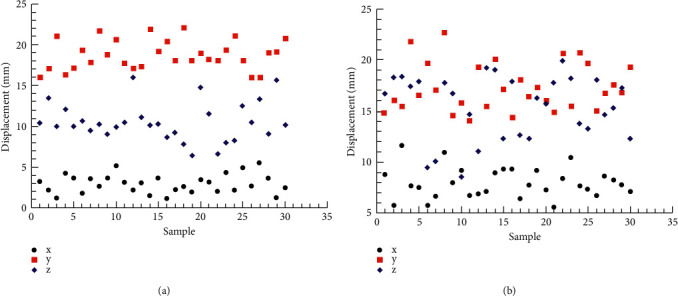
Displacement of papilla in sagittal plane. (a) Flexion 90°. (b) Extension 60°.

Figure 3 shows the displacement of the two breast points of 30 subjects when they perform the test actions of 90° front and 60° back lifting of the upper limbs in the sagittal plane.  Table 3 shows the descriptive statistics (minimum, maximum, mean, and standard deviation) of the breast point raised 90° in front of the sagittal plane and 60° behind the sagittal plane. The results showed that the displacement of the *y*-axis, i.e. the left and right directions, was significantly greater than that of the other axes (18.79 ± 1.77) when the upper limb was lifted 90° before the test, and the largest displacement was also the *y*-axis (17.35 ± 2.33) when the upper limb was lifted 60° after the test. The displacement of the breast point of the *x*-axis was the least in the sagittal plane, which was 2.95 ± 1.15 and 7.94 ± 1.45, respectively.

**Figure 4:**
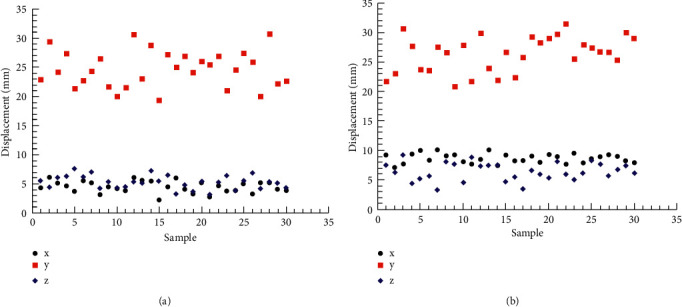
Displacement of transverse papilla. (a) Adduction 135°. (b) Extension 30°.

Figure 4 shows the displacement of the two breast points of 30 subjects when they perform the upper limb internal 135° and external 30° test actions on the transverse plane.  Table 4 shows the descriptive statistics (minimum value, maximum value, mean value, and standard deviation) of milk points at 135° adduction and 30° extension, respectively, on the transverse plane. The results showed that the displacement of the *y*-axis, i.e. the left and right directions, was significantly greater than that of the other axes (24.69 ± 3.09) when the upper limb was retracted at 135°, the largest displacement was also the *y*-axis (26.42 ± 2.95) when the upper limb was extended at 30°, the displacement of the breast point of the upper limb at 135° retracted at the *x*-axis was the least (4.45 ± 0.98) in the sagittal plane, and the displacement of the *z*-axis at 30° was the least (6.41 ± 1.50).

**Figure 5:**
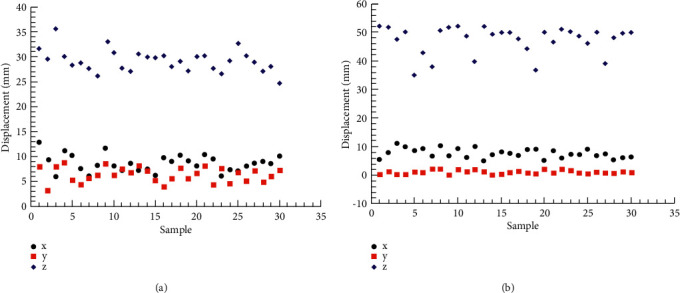
Coronal papillary displacement. (a) Abduction 90°. (b) Abduction 180°.

Figure 5 shows the displacement of two breast points of 30 subjects when performing 90° and 180° upper limb abduction tests on the coronal plane.  Table 5 shows the descriptive statistics (minimum value, maximum value, mean value, and standard deviation) of 90° and 180° abduction of milk points on the transverse plane, respectively. The results showed that when performing 90° and 180° abduction of the upper limb, the displacement of the *z*-axis, i.e. the up-down direction, was significantly greater than that of the other axes, 29.21 ± 2.17 and 47.31 ± 4.86, respectively. The displacement of the breast point of the coronal upper limb movement in the *y*-axis was the least, 6.39 ± 1.45 and 1.06 ± 0.64, respectively.

As shown in Table 6, during the comparative analysis of the breast point on the three sides of the human body during the upper limb movement, it can be seen that the displacement of the breast point in the coronal plane, i.e. the fore-and-aft direction, is the largest, 47.99 ± 4.78 and 31.20 ± 2.27, respectively, while the displacement of the breast point in the sagittal plane, i.e. the left-right direction, is the smallest, 21.88 ± 1.61 and 24.76 ± 2.52, respectively.

As shown in Table 7, there is a weak correlation between the displacement of breast points in each axis between the faces when performing upper limb movements, while the horizontal plane, i.e., 135° adduction in the up and down direction, has a significant correlation with the chest circumference at the level of 0.05, and the displacement caused by other movements has no significant relationship with the chest circumference. In addition, the upper limb movements facing the body have no significant relationship with BMI and lower chest circumference.

### 4.2. Xiphoid Process Displacement Analysis

**Figure 6:**
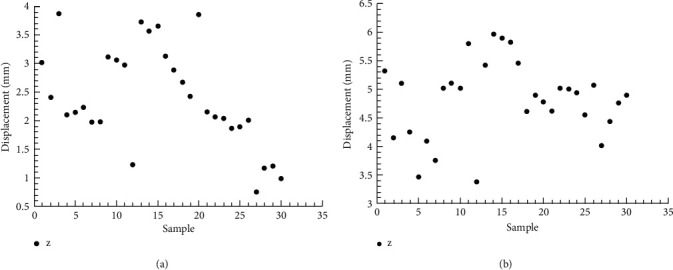
Displacement of xiphoid point in coronal plane. (a)Abduction 90°. (b)Abduction 180°.

Figure 6 shows the displacement of each new measuring point of 30 subjects when performing 90° and 180° upper limb abduction tests on the coronal plane.  Table 8 lists the descriptive statistics (minimum value, maximum value, mean value, and standard deviation) of the xiphoid points at 90° and 180° abduction, respectively, in the transverse plane. The results showed that the displacement of the new measuring point at 180° abduction was greater than that at 90° abduction.

As shown in Table 9, the displacement caused by 90° abduction of the upper limb in the coronal plane and 180° abduction is highly correlated at the level of 0.01, and the displacement is positively correlated with the action amplitude of the upper limb in the coronal axis, but not with BMI, chest circumference, and lower chest circumference.

### 4.3. Comparative Analysis of Displacement between Breast Point and Xiphoid Process

Table 10 provides descriptive statistical information (mean value, range, and standard deviation) by comparing the displacement of the breast point in the sagittal, transverse, and coronal planes and the displacement of the xiphoid point in the coronal plane. The mean value of the displacement of the breast point in the coronal plane is the largest (31.2 ± 12.21 and 47.99 ± 25.76, respectively), and the mean value of the displacement of the xiphoid point is significantly smaller than that of the breast point in the coronal plane (2.41 ± 4.68 and 4.79 ± 13.52, respectively), with high reliability. The displacement range of breast point was the largest when performing coronal plane motion. Therefore, the stability of xiphoid point is higher than that of mammary point.

## 5. Conclusion

In order to verify the stability of xiphoid point as the measuring point of new chest shape, the movement displacement comparison experiment of breast point and xiphoid point was carried out. Through the experiment, it is found that the breast point, as the measuring point of the chest shape, will have a great displacement during the movement of the shoulder joint of the upper limb, which will affect the accuracy of the measurement. Although the xiphoid point has a certain offset due to the attached skin, the overall displacement is far less than that of the breast point, which is less affected by the upper limb movements. It is a progressive chest measurement point, which provides a reference for the innovation and development of chest measurement technology.

In the follow-up research work, based on the new chest measurement point, i.e., xiphoid point, the linear dimension, angular dimension, body surface dimension, concave convex curvature, volume, and other derived dimensions of the breast can be measured to reflect the overall picture of the chest, so as to construct a chest measurement method and measurement tool with high accuracy.

## Figures and Tables

**Table 1:** Age and body stature data of study participants.

Variable	Mean	SD
Age (years)	24.6(21–30)	2.4
Height (m)	1.6(1.55–1.71)	0.0
Weight (kg)	55.5(42–67)	5.4
BMI (kg/m^2^)	21.0(17.5–24.3)	1.8

**Table 2:** Mean ± standard deviation (range) of values characterizing the breasts of the participant group.

Variable	*N* = 30
Chest measurement (cm)	84.2 ± 3.4 (78–90)
Lower chest measurement (cm)	73.8 ± 3.4 (68–82)
Chest circumference difference (cm)	10.4 ± 2.0 (6–15)

**Table 3:** Descriptive statistics of sagittal nipple displacement.

Direction	*N*	SP1				SP2			
		Min	Max	Mean	SD	Min	Max	Mean	SD
*x*	30	1.15	5.56	2.95	1.15	5.56	11.56	7.94	1.45
*y*	30	16.01	22.06	18.79	1.77	14.08	22.76	17.35	2.33
*z*	30	6.43	15.61	10.49	2.28	8.53	19.95	15.43	3.12

**Table 4:** Descriptive statistics of transverse nipple displacement.

Direction	*N*	TP1				TP2			
		Min	Max	Mean	SD	Min	Max	Mean	SD
*x*	30	2.20	6.01	4.45	0.98	7.13	10.11	8.67	0.80
*y*	30	19.37	30.72	24.69	3.09	20.78	31.52	26.42	2.95
*z*	30	3.17	7.56	5.26	1.14	3.24	9.22	6.41	1.50

**Table 5:** Descriptive statistics of nipple displacement in coronal plane.

Direction	*N*	CP1				CP2			
		Min	Max	Mean	SD	Min	Max	Mean	SD
*x*	30	6.04	12.91	8.67	1.68	5.12	11.06	7.67	1.61
*y*	30	3.16	8.76	6.39	1.45	0.18	2.19	1.06	0.64
*Z*	30	24.78	35.66	29.21	2.17	35.12	52.43	47.31	4.86

**Table 6:** Nipple displacement in three directions.

		*N*	Mean	SD
SP	SP1	30	21.88	1.61
	SP2	30	24.76	2.52
TP	TP1	30	25.68	3.13
	TP2	30	28.61	2.74
CP	CP1	30	31.20	2.27
	CP2	30	47.99	4.78

**Table 7:** Analysis of factors affecting nipple displacement.

			SP1	SP2	TP1	TP2	CP1	CP2	BMI	BT	UBT
SP	SP1	Pearson	1	0.022	0.119	0.264	−0.017	0.186	−0.009	0.233	0.126
		Sig.		0.906	0.530	0.159	0.928	0.324	0.960	0.215	0.508
		*F*							0.002	1.020	0.329
	SP2	Pearson	0.022	1	0.350	0.108	−0.028	0.248	0.013	−0.157	−0.125
		Sig.	0.906		0.058	0.571	0.885	0.186	0.945	0.407	0.509
		*F*							0.062	2.990	1.462

TP	TP1	Pearson	0.119	0.350	1	0.089	−0.035	0.024	0.190	0.365^*∗*^	0.162
		Sig.	0.530	0.058		0.639	0.855	0.901	0.314	0.047	0.392
		*F*							1.159	6.141	1.598
	TP2	Pearson	0.264	0.108	0.089	1	−0.098	−0.153	−0.201	−0.070	−0.239
		Sig.	0.159	0.571	0.639		0.606	0.421	0.286	0.713	0.203
		*F*							0.879	0.305	1.240

CP	CP1	Pearson	−0.017	−0.028	−0.035	−0.098	1	0.280	0.145	0.073	0.145
		Sig.	0.928	0.885	0.855	0.606		0.134	0.443	0.700	0.444
		*F*				0			0.326	0.050	0.090
	CP2	Pearson	0.186	0.248	0.024	−0.153	0.280	1	0.108	0.104	0.261
		Sig.	0.324	0.186	0.901	0.421	0.134		0.571	0.586	0.163
		*F*							0.051	0.326	1.368

^*∗*^ 0.05 (two tailed), significant correlation. ^*∗∗*^0.01 (two tailed), significant correlation.

**Table 8:** Descriptive statistics of xiphoid point displacement in coronal plane.

Direction	*N*	CP1				CP2			
		Min	Max	Mean	SD	Min	Max	Mean	SD
*z*	30	0.76	3.87	2.32	0.86	3.37	5.97	4.67	0.66

**Table 9:** Analysis of factors affecting the displacement of xiphoid point.

			CP3	CP4	BMI	BT	UBT
CP	CP3	Pearson	1	0.609^*∗∗*^	−0.036	0.060	0.110
		Sig.		0.000	0.851	0.755	0.563
		F			0.001	0.480	0.173
	CP4	Pearson	0.609^*∗∗*^	1	−0.068	−0.074	0.077
		Sig.	0.000		0.723	0.698	0.687
		*F*			0.090	0.533	0.004

^*∗*^0.05 (two tailed), significant correlation. ^*∗∗*^0.01 (two tailed), significant correlation.

**Table 10:** Comparative analysis of displacement of mastoid point and xiphoid process.

	Breast point				Xiphoid process			
	SP		TP		CP		CP	
	SP1	SP2	TP1	TP2	CP1	CP2	CP3	CP4
Mean	21.88	24.76	25.68	28.61	31.20	47.99	2.41	4.79
R	5.37	10.69	11.52	8.99	8.14	16.77	2.14	8.26
SD	8.65	13.56	16.84	14.76	12.21	25.76	4.68	13.52

## Data Availability

The dataset can be accessed upon request.
